# Hybrid Physics-Informed Residual Learning for Robust BDS-3 Satellite Clock Bias Prediction

**DOI:** 10.3390/s26113475

**Published:** 2026-05-31

**Authors:** Lingfeng Cheng, Keyu Li, Wenhui Guan, Zexian Li, Qin Liang, Chenglin Cai

**Affiliations:** 1 School of Mathematical and Computational Sciences, Xiangtan University, Xiangtan 411105, China; 2 National Center for Applied Mathematics in Hunan, Xiangtan University, Xiangtan 411105, China; 3 School of Automation and Electronic Information, Xiangtan University, Xiangtan 411105, China; 4 Solux College of Architecture and Design, University of South China, Hengyang 421099, China; lzxsements@usc.edu.cn

**Keywords:** PPP, satellite clock bias, BDS-3, PINN, LSTM

## Abstract

**Highlights:**

**What are the main findings?**

- A hybrid physics-informed and data-driven framework integrating PINN and LSTM is proposed for BDS-3 satellite clock bias prediction, effectively separating long-term trend components from short-term residual variations.
- Extensive experiments demonstrate that the proposed PINN-LSTM model consistently outperforms traditional statistical models and purely data-driven deep learning models in both short-term and 24-h clock bias prediction accuracy and stability.

**What are the implications of the main findings?**

- The proposed framework enhances the robustness of real-time satellite clock products under data outages, contributing to improved reliability of RT-PPP applications.
- The hybrid modeling strategy provides a general and extensible paradigm for satellite time series prediction, enabling improved generalization across different satellite clock types and observation periods.

**Abstract:**

Real-time precise point positioning (RT-PPP) has enabled a wide range of high-precision positioning and navigation applications, while its reliability strongly depends on the availability and continuity of precise satellite clock products. In the third-generation BeiDou Navigation Satellite System (BDS-3), interruptions or gaps in real-time precise clock products can significantly degrade the continuity and performance of precise positioning services. Therefore, accurate and robust satellite clock bias (SCB) prediction is essential for supporting reliable RT-PPP applications under product outage conditions. To address this problem, this study proposes a hybrid physics-informed and data-driven framework for BDS-3 SCB prediction. The proposed method sequentially integrates a physics-informed neural network (PINN) and a long short-term memory (LSTM) network. Specifically, the PINN is used to model and extrapolate the physically consistent trend component of SCB increments by embedding clock dynamical constraints through automatic differentiation, while the LSTM is employed to learn and predict the residual sequence containing short-term stochastic variations that cannot be fully captured by the physical model. The final SCB prediction is obtained by reconstructing the trend and residual components and recovering the original clock bias series. The proposed framework is evaluated using BDS-3 precise clock products and compared with conventional models, including quadratic polynomial (QP), autoregressive integrated moving average (ARIMA), convolutional neural network–long short-term memory (CNN-LSTM), and attention-enhanced long short-term memory (LSTM-Attention). Experimental results show that the proposed PINN-LSTM framework consistently achieves superior prediction accuracy and stability at both 12 h and 24 h forecasting horizons. Specifically, compared with QP, ARIMA, CNN-LSTM, and LSTM-Attention, the proposed method improves prediction accuracy by 18.4%, 52.8%, 32.3%, and 33.8%, respectively, for the 12 h forecasting task, and by 34.8%, 58.5%, 41.8%, and 43.8%, respectively, for the 24 h forecasting task. The results further demonstrate reduced long-horizon error accumulation, improved robustness across satellites equipped with different atomic clock types, and stronger generalization across observation days. These findings indicate that the proposed framework can provide effective support for maintaining the continuity and reliability of BDS-3 precise clock products and has strong potential for improving real-time precise positioning applications.

## 1. Introduction

More intelligent and ubiquitous positioning, navigation, and timing (PNT) services have become a central research objective in the field of satellite navigation [1]. As a high-precision positioning technique that does not rely on ground reference stations [2], precise point positioning (PPP) plays a key role in modern PNT systems [3], particularly in real-time applications. At present, real-time PPP (RT-PPP) services based on the third-generation BeiDou Navigation Satellite System (BDS-3) still depend on real-time service (RTS) products provided by institutions such as the International GNSS Service (IGS) [4,5,6,7], including precise satellite orbit and clock products [8]. However, existing precise satellite clock products may suffer from data discontinuities or even complete satellite data outages due to data anomalies, communication interruptions, or network failures [9,10]. Such data gaps, which can last for several hours or longer, severely degrade the accuracy and reliability of RT-PPP solutions [11]. To ensure the robustness of BDS-3 RT-PPP services, it is therefore essential to develop a satellite clock bias (SCB) prediction method capable of providing stable and reliable clock information during periods of data unavailability.

Current SCB prediction techniques can be broadly classified into traditional mathematical modeling approaches and artificial intelligence-based data-driven methods. Traditional SCB prediction methods rely on explicit mathematical formulations to describe clock behavior and have long been used in real-time and near-real-time applications. Polynomial-based models, such as the linear polynomial (LP) and quadratic polynomial (QP) models, approximate SCB evolution using low-order time polynomials [12,13]. The LP model represents clock bias and frequency offset, whereas the QP model further incorporates frequency drift, yielding a simple and physically interpretable description of clock dynamics. These polynomial models are computationally efficient and numerically stable, which makes them suitable for operational use. Their deterministic structure, however, limits adaptability to stochastic disturbances and nonlinear variations, and prediction errors tend to accumulate as the forecasting horizon increases [14,15].

The grey model GM(1,1) suppresses random fluctuations through accumulated generating operations and characterizes SCB evolution using a first-order differential equation, enabling stable short-term prediction under limited data conditions [16,17,18]. Its modeling flexibility is constrained by the predefined exponential form, and prediction accuracy decreases when actual clock behavior deviates from the assumed evolution pattern. Autoregressive integrated moving average (ARIMA) models capture temporal correlations in differenced SCB series and can improve short-term prediction performance under approximately stationary conditions [19,20]. Their applicability is restricted when stationarity assumptions are violated or when clock dynamics vary over time. Spectral analysis models (SAMs) extract dominant periodic components in the frequency domain and perform well when periodic characteristics remain stable [21,22]. Sensitivity to changes in periodic behavior limits their reliability in long-term prediction. Kalman filter-based models formulate SCB prediction within a state–space framework and enable recursive estimation with uncertainty propagation, providing adaptability to short-term variations [23,24]. Prediction performance is strongly influenced by noise modeling strategies and parameter selection. Overall, while traditional models offer clear interpretability and high computational efficiency, their limited ability to jointly represent deterministic trends and complex stochastic behaviors constrains prediction stability under long forecasting horizons or data interruption scenarios.

Artificial intelligence-based approaches formulate SCB prediction as a data-driven sequence learning problem and have attracted increasing attention in recent years. Recurrent neural network (RNN) architectures [25], especially long short-term memory (LSTM) networks [26,27], are widely adopted due to their gated structure, which facilitates learning temporal dependencies and alleviates gradient vanishing issues. LSTM-based models are effective in capturing nonlinear dynamics and short-term variability that are difficult to represent with traditional models. Their prediction performance, however, is strongly influenced by the quantity and representativeness of the available training data. To enhance feature extraction capability, attention-based architectures such as LSTM-Attention introduce adaptive weighting mechanisms that emphasize informative temporal features [28], while hybrid structures such as CNN-LSTM employ convolutional layers to extract local patterns prior to temporal modeling [29]. These designs improve prediction accuracy in many scenarios by strengthening feature representation.

In addition to the above methods, empirical function-based and decomposition-based approaches have also been explored in related geodetic and ionospheric forecasting problems. Representative examples include EEMD-based hybrid forecasting for ionospheric TEC prediction, EOF-based or decomposition-based modeling for global TEC representation, empirical mode decomposition for TEC trend removal and filtering, and decomposition-based denoising for GNSS coordinate time series [30,31,32,33]. These studies indicate that functional decomposition can be useful for separating dominant trend components from more complex residual variations. Compared with such empirical or decomposition-based approaches, the present study employs a physics-informed neural network to obtain a more flexible yet physically constrained trend representation for SCB prediction.

At the same time, increased architectural complexity and reliance on data-driven learning reduce interpretability and robustness under changing data conditions. Transformer-based models further extend sequence modeling capacity through self-attention mechanisms and parallel computation, enabling effective learning of long-range temporal dependencies [34]. Their practical application to SCB prediction is often constrained by the requirement for large training datasets and reduced stability during long-term extrapolation. Overall, although deep learning-based methods demonstrate strong representation power and improved short-term prediction accuracy, the lack of explicit incorporation of physical clock behavior makes them sensitive to data interruptions, distribution shifts, and long-horizon forecasting conditions. This limitation poses challenges for achieving stable, interpretable, and generalizable SCB prediction in operational real-time applications.

To address the aforementioned challenges, this study proposes a hybrid physics-informed and data-driven framework for SCB prediction. The main aim of this work is to enhance prediction accuracy, long-horizon stability, and robustness under data interruption conditions, which are critical for real-time high-precision GNSS applications. The proposed framework integrates a physics-informed neural network (PINN) [35] with a LSTM network in a sequential manner. The PINN is employed to model and extrapolate the physically consistent trend component of SCB variations, while the LSTM focuses on learning and predicting the remaining residual components that are difficult to capture using simplified physical modeling alone. By decoupling trend modeling and residual compensation, the framework improves both prediction stability and interpretability.

The main contributions of this study are summarized as follows:

(1) A hybrid PINN-LSTM framework is developed for BDS-3 satellite clock bias prediction by integrating physics-informed trend modeling with data-driven residual learning in a sequential manner.

(2) A physics-informed modeling strategy is introduced for SCB increments, enabling physically consistent trend extraction and improved long-horizon stability under data interruption conditions.

(3) A comprehensive evaluation is conducted on BDS-3 satellites under different clock types and forecasting horizons, demonstrating the effectiveness and robustness of the proposed framework.

The remainder of this paper is organized as follows. Section 2 introduces the data sources and preprocessing procedures, including data description and preprocessing strategies applied to the BDS-3 SCB series. Section 3 presents the proposed methodology, where the physical modeling of SCB using a PINN, the residual modeling using an LSTM network, and the overall hybrid PINN–LSTM framework are described in detail. Section 4 outlines the experimental setup, including the training strategy and evaluation metrics employed in this study. Section 5 reports the experimental results and analysis, covering both the ablation study and comprehensive comparative performance evaluation. Section 6 provides a discussion of the results, highlighting their implications, limitations, and sources of uncertainty. Finally, Section 7 summarizes the main conclusions and outlines potential directions for future work.

## 2. Data and Preprocessing

### 2.1. Data Description

The BDS-3 precise SCB data used in this study were obtained from the Crustal Dynamics Data Information System (CDDIS) maintained by the National Aeronautics and Space Administration (NASA). The prediction experiments cover a continuous 30-day period from day of year (DOY) 173 to DOY 202 in 2023. The data are sampled at a 30 s interval, resulting in 2880 epochs per day.

After preprocessing, a set of BDS-3 satellites with complete and continuous clock bias records over the entire 30-day period was obtained, as listed in Table 1. According to the onboard atomic clock technology, the selected BDS-3 satellites were further classified into two categories: rubidium atomic clock (Rb) satellites and passive hydrogen maser (PHM) satellites.

### 2.2. Data Preprocessing

Before model construction, the raw SCB series underwent data quality control and transformation procedures, including gross error detection, missing-value repair, first-order differencing, and Min–Max normalization. To ensure the reliability of the experimental dataset, the median absolute deviation (MAD) method was employed to detect and remove abnormal observations in the original clock bias time series [36]. Subsequently, cubic spline interpolation was applied to repair the missing data points.

The original SCB time series generally consists of an initial bias, frequency offset, and frequency drift, which together lead to a pronounced long-term linear or quasi-linear trend. However, the components that critically affect clock bias prediction accuracy are the small-scale nonlinear variations and stochastic fluctuations embedded within these large-scale deterministic trends. If the original SCB series is modeled directly, the learning process must simultaneously capture both the dominant trend and the subtle dynamic variations, which not only increases modeling complexity but also makes it difficult for the model to effectively learn the underlying fine-scale characteristics.

To address this issue, appropriate preprocessing is required to separate the deterministic trend components from the random perturbations. In this study, a first-order differencing scheme, as defined in Equations (1)–(3), is applied to the original clock bias sequence *X*, transforming it into a clock bias increment series Δ*X*.

(1) $$X=[x_{1},x_{2},\ldots ,x_{N}]$$

(2) $$\Delta x_{i}=x_{i+1}-x_{i}$$

(3) $$\Delta X=[\Delta x_{1},\Delta x_{2},\ldots \Delta x_{N-1}]$$

where x*_i_* (*i* = 1, 2, …, N) represents the raw SCB data at the *i*-th epoch, totaling N epochs. Δx*_i_* (*i* = 1, 2, …, N − 1) represents the first-order differenced SCB data at the *i*-th epoch, totaling N − 1 epochs.

Subsequently, the Min–Max normalization method, as expressed in Equations (4) and (5), is applied to the clock bias increment sequence Δ*X* to rescale the data and obtain the normalized clock bias increment sequence *Y*.

(4) $$\Delta {\tilde{x}}_{i}=\frac{\Delta x_{i}-\min\left(\Delta X\right)}{\max\left(\Delta X\right)-\min(\Delta X)}$$

(5) $$Y=[\Delta {\tilde{x}}_{1},\Delta {\tilde{x}}_{2},\ldots ,\Delta {\tilde{x}}_{N-1}]$$

where min(n) and max(n) denote the minimum and maximum values of the sequence n, respectively.

**Figure 1:**
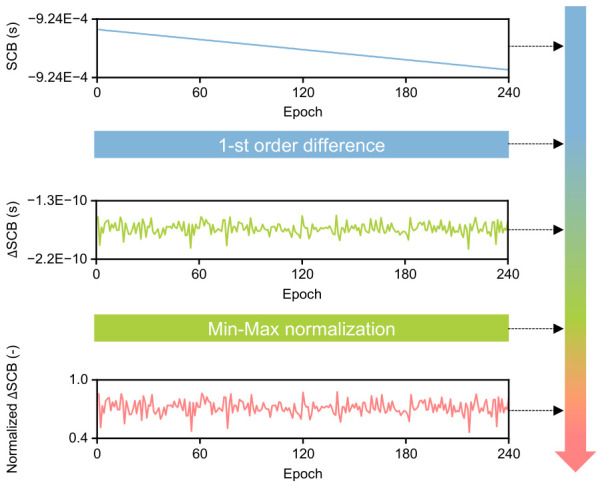
Illustration of SCB data preprocessing, including first-order differencing and normalization. The original SCB and the first-order differenced SCB are expressed in seconds, while the normalized SCB increments are dimensionless.

The preprocessing procedure effectively removes the influence of long-term trends from the original satellite clock bias series and significantly enhances the stationarity of the time series, as illustrated in Figure 1. This transformation improves the numerical stability and generalization capability of the subsequent model training. In addition, the normalized data representation better satisfies the fundamental assumptions underlying both the physics-informed modeling and the data-driven learning stages, thereby providing a solid foundation for improving SCB prediction performance.

## 3. Methodology

To develop a more suitable model for BDS-3 SCB prediction, this study integrates physical constraints with data-driven learning within a unified framework. Specifically, a PINN is employed to model the underlying dynamic behavior of SCB, while a LSTM network is used to predict the residual components not fully captured by the physical model. In the subsequent sections, we provide a detailed analysis of BDS-3 clock bias characteristics and describe the proposed hybrid PINN-LSTM methodology, highlighting how physical interpretability and temporal learning are jointly exploited to improve prediction robustness and accuracy.

### 3.1. Physical Modeling of Clock Bias Using PINN

The physical structure of SCB is fundamentally determined by the intrinsic characteristics of onboard atomic clocks. In time and frequency metrology, the standard dynamical behavior of an atomic clock is commonly modeled as a second-order polynomial function of time, as expressed in Equation (6) [15]. This model has been widely adopted in satellite navigation and time-frequency modeling, providing a physically interpretable description of long-term satellite clock behavior.

(6) $$b\left(t\right)=b_{0}+f_{0}t+\frac{1}{2}dt^{2}+\varepsilon \left(t\right)$$

(7) $$\Delta b\left(t\right)\approx f_{0}+dt+\Delta \varepsilon \left(t\right)$$

(8) $$\frac{d^{2}}{dt^{2}}\Delta b\left(t\right)\approx 0$$

where *b*_0_ denotes the initial clock bias, *f*_0_ represents the frequency offset, *d* corresponds to the frequency drift associated with clock aging, and ε(t) represents stochastic disturbances in the clock behavior.

After applying first-order differencing, the dominant quadratic trend in the original SCB series is largely removed, yielding a clock bias increment sequence that exhibits an approximately linear temporal evolution, as described in Equation (7). Based on this transformation, the second-order time derivative of the clock bias increment, shown in Equation (8), can be reasonably assumed to be close to zero. This assumption is consistent with classical clock state–space models, in which the frequency drift is treated as a slowly varying or nearly constant parameter over short- to medium-term time intervals [24].

Based on the above dynamical assumptions, a PINN is introduced to model the clock bias increment as an explicit function of time. In this study, the PINN takes normalized time as input and outputs the corresponding clock bias increment, while a physics-based regularization term penalizes deviations of the second-order time derivative from zero. This design allows the learned trend component to remain smooth, physically consistent, and suitable for extrapolation beyond the training interval.

Through this formulation, the PINN primarily captures the dominant, physically interpretable trend component of the clock bias increment sequence, providing a stable and well-regularized extrapolation capability beyond the training interval. It should be noted that conventional trend extraction methods, such as polynomial fitting, are also reasonable candidates for modeling the dominant component of SCB evolution. However, in the present study, a PINN was adopted because the objective was not merely to fit a smooth curve, but to incorporate prior clock dynamical knowledge directly into the trend estimation process. By embedding the near-zero second-order derivative constraint into the loss function, the PINN allows the trend component to be learned in a physically consistent and data-adaptive manner. Compared with fixed-form traditional trend models, this formulation provides greater flexibility while preserving physical regularization, which is beneficial for stable trend extrapolation and for the subsequent residual learning stage of the hybrid framework.

**Figure 2:**
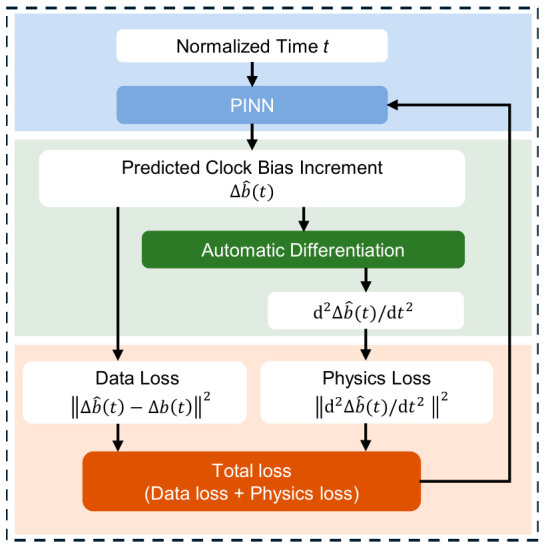
Schematic illustration of the proposed PINN-based clock bias increment modeling framework.

The imposed physical constraint does not aim to fully describe all stochastic or nonlinear behaviors present in the data, but rather to enforce a physically reasonable structure on the learned solution. The overall modeling framework, including the network architecture and the incorporation of the physical constraint, is illustrated in Figure 2.

Unlike purely data-driven fitting approaches, the PINN is designed to capture the physically consistent trend component in the evolution of SCB, rather than reproducing all stochastic fluctuations present in the data. As a result, the PINN provides a smooth and physically interpretable representation of clock bias dynamics, serving as a meaningful baseline for subsequent residual prediction.

### 3.2. Residual Modeling Using LSTM

Although the PINN provides a smooth and physically consistent representation of the dominant trend in SCB increments, it is not designed to explicitly capture all stochastic fluctuations and short-term nonlinear variations inherent in real satellite clock behavior. After removing the physically modeled trend component, the remaining residual sequence primarily reflects effects that are difficult to describe using simplified physical assumptions alone. These variations may include short-term clock instabilities, weakly structured deterministic components, possible periodic or quasi-periodic patterns, nonlinear temporal dependencies, random noise disturbances and potential statistical biases induced by environmental and operational conditions.

To model these residual variations, a LSTM network is adopted as the data-driven correction module. Owing to its capability to capture temporal dependencies in sequential data, LSTM is suitable for learning the remaining short-term structures that are not represented by the physics-informed trend model. In the proposed framework, the LSTM is applied to the residual sequence obtained after PINN-based trend extraction and is used to predict future residual variations through a sliding-window rolling strategy.

The residual sequence is not assumed to be purely irregular or purely stochastic. Rather, it may contain a mixture of remaining temporal structures, including weak deterministic dependencies, possible periodic or quasi-periodic components, nonlinear variations, and stochastic disturbances. The role of the LSTM in this framework is therefore not to uncover explicit physical laws, but to provide a data-driven approximation of residual temporal behavior that remains after physically consistent trend modeling. Through its recurrent structure and memory capability, the LSTM can effectively capture these residual temporal dependencies and improve prediction accuracy beyond the physics-informed trend component alone.

**Figure 3:**
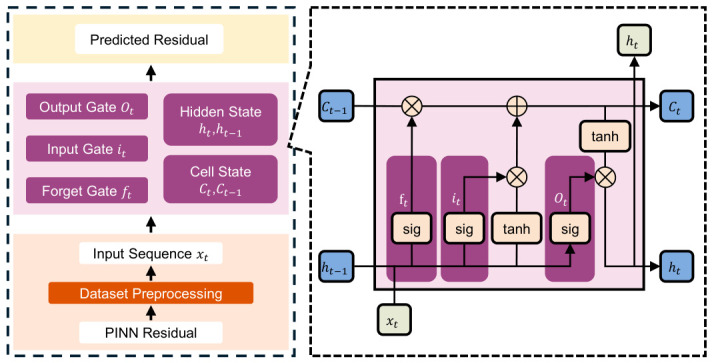
Residual modeling framework based on PINN and LSTM, the left panel shows the residual prediction workflow, while the right panel provides a schematic illustration of the LSTM cell. In the right panel, $x_{t}$ denotes the input at epoch t; $i_{t}$, $f_{t}$, and $o_{t}$ denote the input, forget, and output gates, respectively; $h_{t}$ denotes the hidden state; and $c_{t}$ denotes the cell state.

Figure 3 illustrates the overall residual modeling framework, along with a schematic representation of the LSTM cell adopted in this study. The LSTM does not directly predict the SCB itself. Rather, it functions as a correction module that complements the physically informed PINN by compensating for residual variations. The final SCB prediction is obtained only after recombining the PINN-derived trend component with the LSTM-predicted residuals, ensuring both physical consistency and enhanced predictive flexibility.

### 3.3. Hybrid PINN-LSTM Framework

**Figure 4:**
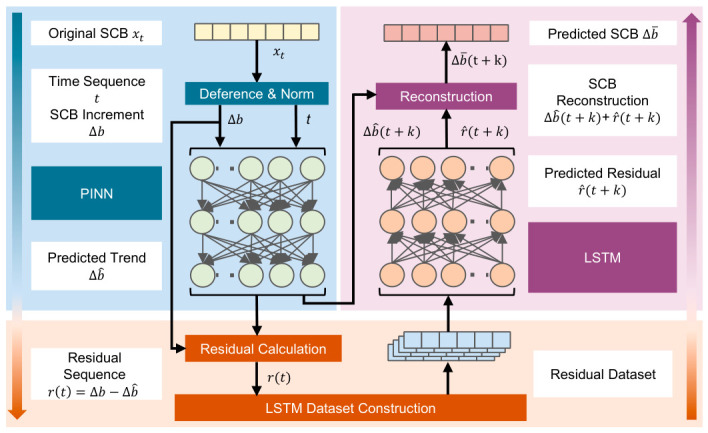
Overall framework of the proposed hybrid PINN-LSTM model for SCB prediction, $\Delta \hat{b}$ denotes the trend component predicted by the PINN, $\hat{r}$ represents the residual component predicted by the LSTM, and $\Delta \bar{b}$ indicates the final SCB increment prediction obtained by combining the predicted trend and residual components.

The overall framework of the proposed hybrid PINN-LSTM method for SCB prediction is illustrated in Figure 4. The proposed approach integrates physics-informed modeling and data-driven learning in a sequential and complementary manner, aiming to jointly exploit physical consistency and temporal dependency in SCB time series. By explicitly separating trend modeling from residual correction, the framework is designed to balance interpretability, flexibility, and predictive robustness.

After data preprocessing, the normalized time sequence together with the corresponding SCB increment series are first used to train the PINN. In this stage, the PINN focuses on learning the dominant evolution pattern of SCB increments by embedding clock dynamical characteristics into the training process through physics-based constraints. As a result, the PINN produces a smooth and physically consistent estimation of the trend component, which represents the primary time-dependent behavior of the SCB increments. This modeling strategy allows the PINN to provide both accurate in-sample fitting and stable extrapolation of the trend over the forecasting horizon.

Once the trend component is obtained, a residual sequence is constructed by subtracting the PINN-predicted trend from the observed SCB increments in the training set. This residual sequence primarily reflects temporal variations that are difficult to represent using simplified physical modeling alone, including weakly structured deterministic components, possible periodic or quasi-periodic patterns, short-term nonlinear fluctuations, stochastic disturbances, and other unmodeled influences. By isolating these components, the complexity of the learning task is significantly reduced for the subsequent data-driven model.

The residual sequence is then reorganized into a supervised learning dataset and modeled using a LSTM network. A sliding-window strategy is adopted to capture local temporal dependencies and enable multi-step rolling prediction of future residual components. Benefiting from its gated recurrent structure, the LSTM is able to learn complex short-term temporal patterns and compensate for the limitations of the physical trend model without directly predicting the SCB itself.

Finally, the predicted residuals are combined with the PINN-derived trend predictions to reconstruct future SCB increments. These predicted increments are subsequently accumulated to recover the final SCB predictions in the original domain. Through this sequential reconstruction process, the proposed framework preserves the physical interpretability of the trend component while enhancing overall prediction accuracy through data-driven residual correction.

By integrating physics-informed trend modeling with data-driven residual learning, the hybrid PINN-LSTM framework effectively reduces error accumulation, improves robustness against data interruptions, and enhances generalization across different satellites and forecasting horizons. This complementary design enables the proposed method to achieve stable and accurate SCB prediction under practical real-time application conditions.

## 4. Experimental Setup

To improve clarity of the experimental design, the main models considered in this study are summarized in Table 2. All experiments were implemented using the PyTorch (version 1.9.1) library and conducted on a workstation equipped with an NVIDIA RTX 4090D GPU. The SCB data used in this study were obtained from the CDDIS, with a sampling interval of 30 s, covering the period from DOY 173 to DOY 202 in 2023.

The hyperparameters adopted in this study were determined empirically through controlled preliminary sensitivity experiments and were then fixed for all reported comparative experiments. In particular, the training length was selected by comparing candidate settings of 0.5, 1, 2, 3, and 4 days on six representative satellites while keeping the other hyperparameters unchanged, and choosing the setting that yielded the most favorable RMSE performance. The remaining hyperparameters were determined in the same manner. To ensure a fair comparison, all baseline models were implemented using consistent data preprocessing strategies and comparable parameter settings, following commonly accepted configurations reported in the literature.

### 4.1. Training Strategy

The proposed method is trained using 48 h of SCB increment data, corresponding to 5760 epochs, and employs a sliding-window prediction strategy based on the last window of the training set to perform rolling forecasts of SCB increments over a 24-h horizon [28]. The predicted increment series is then accumulated to reconstruct the SCB at each epoch. For the formal experiments reported in this study, the selected training length was used for model fitting, and the subsequent 24 h were used for forecasting evaluation. No dedicated validation subset was introduced.

The main hyperparameter configurations of the PINN-LSTM model are summarized in Table 3. The PINN is trained using a full-batch strategy during the physical trend modeling stage to ensure temporal continuity of the imposed physical constraints. The LSTM model incorporates an adaptive early-stopping mechanism (patience = 50) during training to mitigate overfitting.

### 4.2. Evaluation Metrics

To quantitatively evaluate the prediction performance of different models, the root mean square error (RMSE), mean absolute error (MAE), and the 95th percentile error (P95) are adopted as evaluation metrics, as defined in Equations (9)–(11), respectively. RMSE measures the overall magnitude of prediction errors and is sensitive to large deviations, while MAE reflects the average absolute prediction error and provides a more robust assessment against outliers. The P95 metric characterizes the robustness of the prediction by indicating the upper bound within which 95% of the absolute errors fall.

(9) $$\mathrm{RMSE}=\sqrt{\frac{1}{2}{\sum_{t=1}^{T}\left({\hat{y}}_{t}-y_{t}\right)}^{2}}$$

(10) $$\mathrm{MAE}=\frac{1}{T}\sum_{t=1}^{T}\left|{\hat{y}}_{t}-y_{t}\right|$$

(11) $$\mathrm{Pr}\left(\left|{\hat{y}}_{t}-y_{t}\right|\leq P95\right)=0.95$$

where *t* = 1, 2, …, T indicates the epoch index over the forecasting horizon, ${\hat{y}}_{t}$ and $y_{t}$ denote the predicted and observed SCB values at epoch *t*, respectively, and Pr(⋅) denotes the percentile operator. The P95 error represents the upper bound of the absolute prediction error distribution excluding the largest 5% of deviations.

For each prediction day and each satellite, the above metrics are computed over the entire 24-h forecasting period to evaluate daily prediction accuracy and satellite-specific performance. In addition, horizon-dependent error metrics are calculated for prediction horizons ranging from 1 to 24 h, allowing an analysis of error growth behavior as the forecasting horizon increases. This evaluation framework enables a comprehensive comparison of different models in terms of prediction accuracy, robustness, and long-term stability. For clarity of model comparison, the reported RMSE, MAE, and P95 values are presented to four decimal places. This presentation is intended to preserve small numerical differences among closely competing models in the aggregated statistics, rather than to imply absolute physical significance at the same decimal level.

## 5. Results and Analysis

### 5.1. Ablation Study

To verify the necessity and effectiveness of individual components in the proposed hybrid modeling strategy, an ablation study was conducted by comparing the proposed PINN-LSTM model with its two constituent variants, namely a purely physics-informed PINN model and a purely data-driven LSTM model. This subsection is intended to examine the contribution of trend modeling and residual learning within the proposed framework, rather than to provide a benchmark comparison against external baseline methods. Considering the differences in onboard atomic clock types, six BDS-3 satellites were selected for evaluation, including three Rb clock satellites (C32, C33, and C36) and three PHM clock satellites (C25, C26, and C27). The detailed results are summarized in Table 4, Table 5 and Table 6.

The results summarized in Table 4, Table 5 and Table 6 present a comparative ablation analysis of the LSTM, PINN, and PINN-LSTM models under 12-h and 24-h prediction horizons for the selected BDS-3 satellites. Overall, the hybrid PINN-LSTM model yields lower prediction errors than the standalone LSTM model across all satellites and evaluation metrics at both forecasting horizons.

When compared with the PINN model, the PINN-LSTM approach achieves comparable or improved performance for most satellites and metrics, particularly at the 24-h prediction horizon. In several 12-h prediction cases, the error levels of PINN-LSTM and PINN are close, indicating that the physics-informed trend component already provides a strong short-term representation. However, as the prediction horizon extends, the advantage of incorporating residual modeling through LSTM becomes more evident. As expected, prediction errors increase for all models when the forecasting horizon is extended from 12 h to 24 h, reflecting the growing difficulty of long-term SCB prediction. Nevertheless, the PINN-LSTM model consistently exhibits a smaller increase in RMSE, MAE, and P95 values across satellites, indicating a reduced rate of error accumulation. In particular, at the 24-h horizon, the PINN-LSTM model achieves the lowest or near-lowest RMSE, MAE, and P95 values for the majority of the evaluated satellites. The clock type-specific average results further confirm that the hybrid model provides stable and improved performance for both Rb and PHM satellites in both short-term and long-term prediction scenarios. The improvement is particularly evident for the Rb group, while the model also maintains competitive performance for the PHM group. These results demonstrate that integrating physics-informed trend modeling with data-driven residual learning effectively enhances robustness and mitigates long-horizon degradation in SCB prediction.

### 5.2. Comparative Performance Evaluation

To comprehensively evaluate the characteristics and advantages of the proposed PINN-LSTM model for BDS-3 SCB prediction, this subsection presents a benchmark comparison against external baseline methods, including two traditional models, namely QP and ARIMA, as well as two representative deep learning approaches, CNN-LSTM and LSTM-Attention. Unlike the ablation study in Section 5.1, which focuses on the contribution of the internal components of the proposed framework, the present subsection is intended to assess the overall performance of the proposed method against commonly used baseline models. All models are applied to the complete set of 22 BDS-3 satellites listed in Table 1, ensuring a fair and consistent comparison under identical experimental conditions.

To analyze the error accumulation behavior over a 24-h forecasting horizon, the RMSE, MAE, and P95 metrics are computed for the prediction results of all satellites. These metrics are then averaged across satellites for each model. This analysis provides an intuitive comparison of different models in terms of prediction accuracy, robustness, and long-term stability as the forecasting horizon increases.

**Figure 5:**
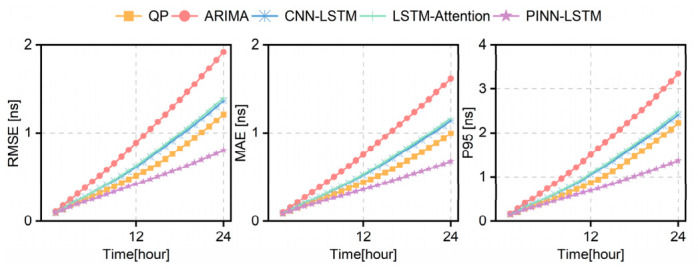
Comparison of the 24-h evolution of RMSE, MAE, and P95 averaged over all BDS-3 satellites for different prediction models, with different colors and markers representing different models.

To provide a quantitative comparison, the RMSE, MAE, and P95 metrics at the 12-h and 24-h prediction horizons are summarized in Table 7. In addition, the temporal evolution of these metrics over the 24-h forecasting period is illustrated in Figure 5. By jointly examining Table 7 and Figure 5, the ARIMA model consistently exhibits larger errors across all three metrics. As the prediction horizon increases, the RMSE, MAE, and P95 values of ARIMA grow at a noticeably faster rate than those of the other models. The two data-driven deep learning models, CNN-LSTM and LSTM-Attention, show comparable prediction performance, while their error accumulation rates are substantially lower than that of ARIMA. The QP model exhibits slightly slower error growth than the deep learning models over the forecasting horizon.

In contrast, among all evaluated models, the proposed PINN-LSTM approach achieves the lowest RMSE, MAE, and P95 values at both the 12-h and 24-h prediction horizons. Furthermore, the error growth curves in Figure 5 indicate that the increase in prediction errors for PINN-LSTM is more gradual compared with the other methods, suggesting reduced error accumulation over longer forecasting intervals.

**Figure 6:**
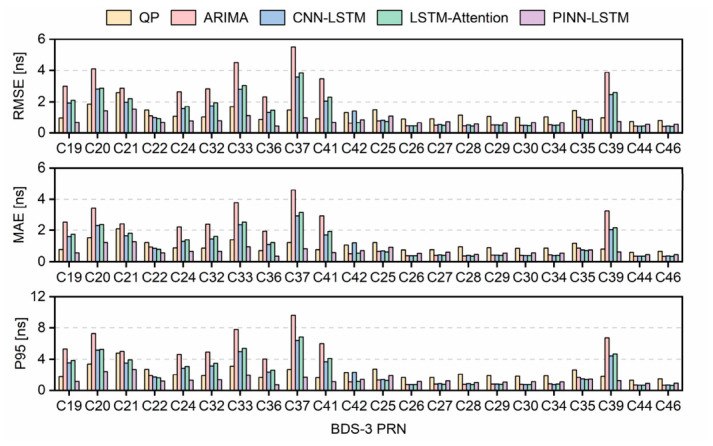
Satellite-wise comparison of 24-h averaged RMSE, MAE, and P95 for different prediction models. Different colors denote different models. The satellites are grouped by onboard atomic clock type and ordered by PRN number within each group, as listed in Table 1. Specifically, C25–C30, C34–C35, C39, C44, and C46 are PHM satellites, while the remaining satellites shown are Rb satellites.

To further examine the satellite-specific performance of different prediction models, Figure 6 presents bar charts of the 24-h averaged RMSE, MAE, and P95 for each BDS-3 satellite. As BDS-3 satellites are equipped with different atomic clock technologies, the magnitude of clock drift varies across satellites, which leads to heterogeneous prediction performance among different clock types [12]. It can be observed that all models tend to exhibit relatively larger prediction errors for most Rb clock satellites, while achieving comparatively better performance for most PHM clock satellites. In particular, the ARIMA, CNN-LSTM, and LSTM-Attention models generally show higher RMSE, MAE, and P95 values on Rb satellites than the QP and PINN-LSTM models, whereas an opposite tendency is observed for most PHM satellites. The QP model demonstrates slightly better performance on Rb satellites compared with ARIMA and deep learning-based models, but shows relatively inferior performance on PHM satellites. These results indicate that both purely statistical models and purely data-driven deep learning models exhibit limited generalization capability across satellites with different clock characteristics. Their prediction performance is sensitive to clock type, leading to inconsistent behavior between Rb and PHM satellites.

In contrast, by integrating physics-informed constraints with data-driven residual learning, the proposed PINN-LSTM model effectively mitigates the impact of large clock drift and improves prediction robustness. As shown in Table 7 and Figure 6, PINN-LSTM not only achieves superior overall prediction accuracy, but also exhibits the smallest performance variation between Rb and PHM satellites, suggesting improved adaptability under different clock conditions.

**Figure 7:**
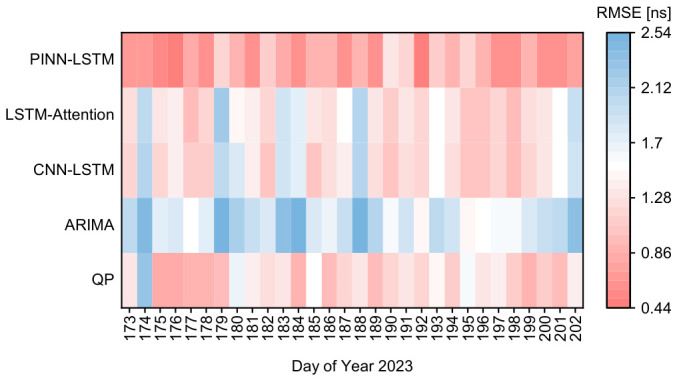
Heatmap of daily averaged RMSE values for different prediction models over DOY 173–202. Each column represents one DOY, each row represents one prediction model, and the RMSE values are averaged over all BDS-3 satellites for that day. The color scale represents the magnitude of the RMSE values, with color variation indicating differences in prediction error levels.

To investigate the generalization performance and robustness of different models across different observation days, a heatmap of the daily averaged RMSE values from DOY 173 to DOY 202 is presented in Figure 7. The RMSE values are computed by averaging the prediction errors of all BDS-3 satellites for each day. The numerical ranges discussed below are based on the statistical distribution of these daily averaged RMSE values.

As illustrated in Figure 7, the ARIMA model exhibits consistently poor prediction performance on almost all observation days, with daily averaged RMSE values remaining above approximately 1.28 ns throughout the evaluation period. The QP, CNN-LSTM, and LSTM-Attention models demonstrate moderate performance, with daily RMSE values generally concentrated in the range of 1.2–1.5 ns. Among these methods, the QP model shows slightly improved stability, as no pronounced high-error days are observed. Nevertheless, both traditional models and purely data-driven deep learning models exhibit relatively large day-to-day fluctuations in RMSE, indicating limited robustness to inter-day variability. In contrast, the proposed PINN-LSTM model achieves the lowest RMSE values on most days, with the majority of daily averaged RMSE values remaining below approximately 0.86 ns and exhibiting comparatively smaller variations across days. These results suggest that integrating physics-informed constraints with data-driven residual learning enables more stable prediction performance under varying daily conditions, thereby significantly enhancing the cross-day generalization capability of BDS-3 SCB prediction.

**Figure 8:**
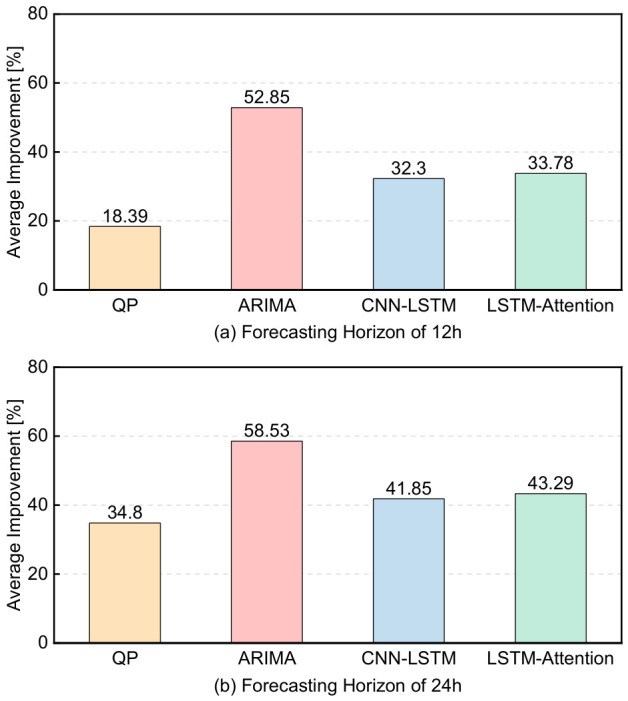
Average performance improvement of the proposed PINN-LSTM model over the baseline methods at different prediction horizons: (**a**) 12-h prediction horizon and (**b**) 24-h prediction horizon. The compared baseline models are QP, ARIMA, CNN-LSTM, and LSTM-Attention, and the reported improvements are evaluated in terms of RMSE, MAE, and P95.

Figure 8 summarizes the average performance improvements achieved by the proposed PINN-LSTM model over the QP, ARIMA, CNN-LSTM, and LSTM-Attention models in terms of RMSE, MAE, and P95 metrics. For the 12-h prediction horizon, the proposed model improves overall prediction accuracy by 18.4%, 52.8%, 32.3%, and 33.8%, respectively, compared with the four baseline models. When the prediction horizon is extended to 24 h, the corresponding improvements further increase to 34.8%, 58.5%, 41.8%, and 43.8%. These results indicate that the hybrid physics-informed and data-driven PINN-LSTM framework consistently outperforms both traditional mathematical models and purely data-driven deep learning approaches across all evaluation metrics. Moreover, the relative performance gains become more pronounced as the prediction horizon increases, demonstrating the superior capability of the proposed method in mitigating long-term error accumulation and maintaining robust prediction accuracy.

## 6. Discussion

This study develops a hybrid physics-informed and data-driven framework for BDS-3 SCB prediction by sequentially integrating a PINN with a LSTM network. The core design of this framework is motivated by the mixed nature of SCB dynamics, which consist of a smooth dominant component primarily governed by clock drift-related behavior and a residual component influenced by short-term instabilities and unmodeled effects.

By transforming the original SCB series into first-order increments, long-term deterministic trends are effectively suppressed, resulting in a representation that is more suitable for learning temporal dynamics. Within this formulation, the PINN explicitly embeds prior clock dynamical knowledge through automatic differentiation, enabling physically consistent modeling and extrapolation of the smooth trend component. The residual sequence, obtained by removing the PINN-estimated trend, mainly reflects the remaining temporal variations not captured by the physics-informed trend model, including weakly structured deterministic components, possible periodic or quasi-periodic patterns, short-term nonlinear fluctuations, stochastic disturbances, and statistical variations associated with environmental and operational changes. These residual components are subsequently modeled using an LSTM network under a sliding-window rolling prediction strategy.

This decoupled modeling strategy alleviates the burden on a single purely data-driven model to simultaneously capture both long-term evolution and short-term variability. The PINN provides a physically interpretable representation of the underlying SCB evolution, allowing the LSTM to focus on compensating for the remaining complex temporal dependencies. Experimental results demonstrate that, compared with traditional statistical models and purely data-driven deep learning approaches, the proposed framework achieves improved prediction accuracy and enhanced stability, with particularly pronounced performance gains observed for BDS-3 satellites equipped with rubidium atomic clocks. These results suggest that incorporating physical consistency into the learning process can improve robustness against inter-satellite and inter-day variability.

Nevertheless, several limitations of the current study should be acknowledged. First, the reliance on rolling multi-step prediction for residual forecasting inevitably introduces error propagation, which may dominate performance over extended prediction horizons. Second, although the adopted physical prior supports stable trend learning, it remains a simplified representation and may not fully capture more complex phenomena, such as temperature-dependent effects, satellite maneuvers, or intermittent clock anomalies. In addition, since precise satellite clock products are estimated quantities, their quality may also be affected under disturbed space weather conditions, such as geomagnetic storms, which could further influence the robustness of SCB prediction in practical applications. The present study did not separately assess such disturbed conditions. The experimental evaluation is restricted to BDS-3 satellites, and further validation across longer time spans and more diverse operational conditions is required to fully assess generalization capability.

## 7. Conclusions

This study proposes a hybrid PINN-LSTM framework for satellite clock bias prediction of the BDS-3 constellation, integrating physics-informed trend modeling with data-driven residual learning in a sequential manner. By separating physically consistent trend estimation from residual compensation, the proposed approach improves prediction accuracy, long-horizon stability, and robustness under data interruption scenarios.

Comprehensive experiments demonstrate that the proposed framework consistently outperforms conventional statistical models and purely data-driven deep learning methods in both short-term and long-term prediction tasks, while also providing enhanced interpretability. The results indicate that combining physical information with data-driven learning offers an effective pathway for improving satellite clock bias prediction, particularly for applications requiring reliable real-time performance.

Future work will focus on mitigating error accumulation in rolling prediction, developing more adaptive physical constraints and residual modeling strategies, and extending the proposed framework to other GNSS constellations, including GPS, Galileo, and GLONASS. Overall, the PINN-LSTM framework provides a promising and interpretable solution for enhancing the robustness of real-time precise positioning services when high-quality precise products are unavailable or degraded.

## Figures and Tables

**Table 1:** BDS-3 satellites information (DOY 173 to 202, 2023).

Clock	PRN
Rb	C19, C20, C21, C22, C24, C32, C33, C36, C37, C41, C42
PHM	C25, C26, C27, C28, C29, C30, C34, C35, C44, C39, C46

**Table 2:** Summary of the models considered in this study.

Model	Category	Role in This Study	Brief Description
QP	Traditional model	Baseline	Quadratic polynomial model used to describe SCB evolution with bias, frequency offset, and drift terms.
ARIMA	Traditional model	Baseline	Statistical time-series model that captures temporal correlations in differenced SCB series.
CNN-LSTM	Deep learning model	Baseline	Hybrid deep learning model that combines convolutional feature extraction with temporal sequence modeling.
LSTM-Attention	Deep learning model	Baseline	LSTM-based model enhanced by an attention mechanism for adaptive temporal feature weighting.
LSTM	Deep learning model	Ablation model	Purely data-driven residual prediction model used as an internal comparison in the ablation study.
PINN	Deep learning model	Ablation model	Trend modeling model that incorporates physical constraints into the learning process.
PINN-LSTM	Deep learning model	Proposed method	Proposed framework combining PINN-based trend extraction with LSTM-based residual prediction.

**Table 3:** Hyperparameter configuration of the PINN-LSTM model.

Parameter	PINN	LSTM
Hidden layer	3	2
Hidden units	64, 64, 64	32, 32
Activation function	tanh	tanh
Learning rate	0.001	0.00001
Optimizer	Adam	Adam
Loss function	mean squared error	mean squared error
Batch size	N/A	64
Input window length	N/A	480
Training epochs	3000	adaptive (early-stopping)

**Table 4:** Ablation comparison of RMSE (ns) for LSTM, PINN, and PINN-LSTM on selected BDS-3 satellites under 12-h and 24-h prediction horizons.

PRN	12-h			24-h		
	LSTM	PINN	PINN-LSTM	LSTM	PINN	PINN-LSTM
C32	1.2659	0.4034	**0.3004**	2.6100	0.5540	**0.2270**
C33	0.9710	0.5383	**0.4720**	3.1539	0.4659	**0.3706**
C36	0.6307	**0.2748**	0.2869	1.5190	0.5883	**0.5664**
C25	0.6374	0.5254	**0.5036**	1.2834	0.9935	**0.9488**
C26	0.4660	0.5930	**0.4470**	0.6875	0.9501	**0.6540**
C27	0.1678	0.1587	**0.1251**	0.3789	0.3485	**0.2620**
Rb Mean	0.9559	0.4055	**0.3531**	2.4276	0.5361	**0.3880**
PHM Mean	0.4237	0.4257	**0.3586**	0.7833	0.7640	**0.6216**

**Table 5:** Ablation comparison of MAE (ns) for LSTM, PINN, and PINN-LSTM on selected BDS-3 satellites under 12-h and 24-h prediction horizons.

PRN	12-h			24-h		
	LSTM	PINN	PINN-LSTM	LSTM	PINN	PINN-LSTM
C32	1.1590	0.3829	**0.2609**	2.2684	0.5183	**0.1746**
C33	0.7751	0.4930	**0.4342**	2.4814	0.4304	**0.3164**
C36	0.5651	**0.2245**	0.2351	1.2664	0.4993	**0.4873**
C25	0.5461	0.4620	**0.4464**	1.0955	0.8598	**0.8224**
C26	0.4013	0.5109	**0.3846**	0.6182	0.8462	**0.5882**
C27	0.1393	0.1307	**0.0958**	0.3157	0.2908	**0.2144**
Rb Mean	0.8331	0.3668	**0.3101**	2.0054	0.4827	**0.3261**
PHM Mean	0.3622	0.3679	**0.3089**	0.6765	0.6656	**0.5417**

**Table 6:** Ablation comparison of P95 (ns) for LSTM, PINN, and PINN-LSTM on selected BDS-3 satellites under 12-h and 24-h prediction horizons.

PRN	12-h			24-h		
	LSTM	PINN	PINN-LSTM	LSTM	PINN	PINN-LSTM
C32	1.9557	0.5785	**0.5322**	4.4998	0.8599	**0.4962**
C33	1.8087	0.7754	**0.6704**	5.8953	0.7456	**0.6486**
C36	1.0760	**0.4547**	0.4690	2.8824	0.9273	**0.8806**
C25	1.0142	0.8247	**0.7876**	2.1543	1.6759	**1.6102**
C26	0.7506	0.9149	**0.7259**	1.0536	1.4920	**1.0036**
C27	0.2896	0.2771	**0.2334**	0.6555	0.6053	**0.4714**
Rb Mean	1.6135	0.6029	**0.5572**	4.4258	0.8443	**0.6751**
PHM Mean	0.6848	0.6722	**0.5823**	1.2878	1.2577	**1.0284**

**Table 7:** Mean RMSE, MAE, and P95 (ns) of different models for 12-h and 24-h SCB prediction, averaged over all BDS-3 satellites.

PRN	12-h			24-h		
	RMSE	MAE	P95	RMSE	MAE	P95
QP	0.5152	0.4329	0.8683	1.2150	0.9943	2.2257
ARIMA	0.8844	0.7532	1.5068	1.9231	1.6185	3.3479
CNN-LSTM	0.6156	0.5221	1.0554	1.3694	1.1394	2.4235
LSTM-Attention	0.6292	0.5324	1.0823	1.4039	1.1674	2.4879
PINN-LSTM	**0.4203**	**0.3596**	**0.6961**	**0.7999**	**0.6781**	**1.3702**

## Data Availability

The experimental data in the manuscript are all public data and can be downloaded from https://cddis.nasa.gov/archive (accessed on 25 May 2026).The SCB data used in this study were obtained from the CDDIS, with a sampling interval of 30 s, covering the period from DOY 173 to DOY 202 in 2023.
